# Association between parity and macrosomia in Shaanxi Province of Northwest China

**DOI:** 10.1186/s13052-020-0784-x

**Published:** 2020-02-18

**Authors:** Fangliang Lei, Lili Zhang, Yuan Shen, Yaling Zhao, Yijun Kang, Pengfei Qu, Baibing Mi, Shaonong Dang, Hong Yan

**Affiliations:** 1 Department of Epidemiology and Health Statistics, School of Public Health, Xi’an Jiaotong University Health Science Center, Xi’an, 710061 Shaanxi China; 2 Shaanxi Provincial People’s Hospital, Xi’an, 710068 Shaanxi China; 3 Assisted Reproduction Center, Northwest women and children’s Hospital, Xi’an, 710003 Shaanxi China; 4 Xi’an Jiaotong University, Health Science Center, Xi’an, 710061 Shaanxi China; 5 Nutrition and Food Safety Engineering Research Center of Shaanxi Province, Xi’an, 710061 Shaanxi China; 6 Key Laboratory of Environment and Genes Related to Diseases (Xi’an Jiaotong University), Ministry of Education, Xi’an, 710061 Shaanxi China

**Keywords:** Parity, Macrosomia, Association, Primipara, Multipara

## Abstract

**Objective:**

To explore the relationship between parity and macrosomia and provide the necessary reference for the maternal and children health service.

**Method:**

A cross-sectional epidemiological survey with the purpose to assess the birth outcomes was conducted in Shaanxi province, China.

**Results:**

The incidence of macrosomia in multiparas was higher than that in primiparas. Univariate analysis showed that maternal age < 25 years, peasant/housework, living in rural areas and female infants were the protective factors of macrosomia. The possibility of having a macrosomic infant also increased with gestational age, maternal education level, household wealth index, living in Central Shaanxi and gestational diabetes. The generalized linear mixed models represented the association between parity and macrosomia. After adjusting for statistically significant factors in univariate analysis from model 1 to model 3, the risk of being born macrosomia was 1.26 times higher for a multipara compared to that for a primipara.

**Conclusions:**

Present study indicated parity of two children was associated with increased risk for macrosomic births compared with parity of one child. Compared to primiparas, multiparas should far strengthen the pre-pregnancy education and the guidance during pregnancy to control pre-pregnancy body mass index and pregnancy weight, and keep the appropriate exercise and balanced diet.

## Key-points

- We found that parity was related to the occurrence of macrosomia. The incidence of macrosomia of multipara was higher than primipara, and the difference was statistically significant. Compared to primiparas, multiparas should far strengthen the pre-pregnancy education and the guidance during pregnancy to control pre-pregnancy body mass index and pregnancy weight, and keep the appropriate exercise and balanced diet.
- After adjusting for statistically significant factors in univariate analysis, analysis based on generalized linear mixed models revealed that the risk of macrosomia was 1.26times higher for a secondborn child compared to a first born.
- The primary strength of the present analysis is the large sample size (27,351 single live births occurring from 2010 to 2013), which accounted for ~ 9% of neonates in Shaanxi Province. Therefore, our results can be generalized to the entire province as well as Northwest China.

## Introduction

Birth weight (BW) is an important indication of mothers’ and neonates’ nutritional status, and may be the important determinant of infant’s survival and future health, growth, and development [1]. Macrosomia is defined as birth weight greater than or equal to 4.0 kg [2–4]. Macrosomia prevalence in developed countries is between 5 and 20%, although an increase of 15 to 25% has been reported in the past decades. With rapid economic growth in China in the past three decades, investments in education, healthcare and sanitation have increased accordingly. Chinese national health services survey showed that birth weight increased from 3186 g in 1993 to 3284 g in 1998 and to 3307 g in 2003 [5]. A rapid increase in rate of macrosomia has been reported in China. For example, one study about secular trends of macrosomia in southeast China reported an increase from 6.0% in 1994 to 7.8% in 2005 [2]. In Shanghai, the rates of macrosomia increased by 50% between 1989 and 1999.

Maternal complications of macrosomia include prolonged labor, labor augmentation with oxytocin, cesarean delivery, postpartum hemorrhage, infection, 3rd- and4^rd^-degree perineal tears, thromboembolic events and anesthetic accidents [6, 7]. According the American College of Obstetricians and Gynecology (ACOG) practice bulletin macrosomic fetuses have a greater risk for perinatal asphyxia, meconium aspiration, clavicular fracture, brachial plexus injury, and shoulder dystocia [8]. Furthermore, macrosomic infants are at an increased risk of type 2 diabetes mellitus, hypertension, and obesity in adulthood [9–14].

Maternal parity is a well-recognized predictor of infant birthweight, with the lowest birthweights observed among infants born to nulliparous women [15–20]. Birthweight differences across parity have also been shown in prior sibling analyses [15–20]. One prior study reported a birthweight difference of 118 g between first and second born infants; however, when limited only to sibling pairs the difference was even greater at 138 g [21]. Most prior studies focused on the association between parity and birthweight [21, 22]. In addition, several studies have reported that the multiparity is one of the risk factors for macrosomia, or explained the association between parity and macrosomia [23–25]. However, few studies from China were performed for the association between parity and macrosomia. A large population-based sampling survey which was conducted in Shaanxi province of Northwest China to assess birth outcomes allowed us to study the relationship parity and macrosomia.

## Materials and methods

### Study design and participants

The cross-sectional study was executed in Shaanxi province of Northwest China from August to November 2013. The infants born during 2010–2013 and their mothers were the objects of the research. Because of the different population density and fertility rates between rural and urban areas in the whole province, a hierarchical, polystage, probability-proportional-to-size sampling method was used in the present research. In China, administrative organization was divided into 3-hierarchy frames. Counties, townships and villages constitute the rural areas. Independent of rural areas, districts, streets and communities constitute the urban areas. In the first place, 20 counties and 10 districts were randomly selected from the whole province. Then, six townships and three streets were randomly sampled in the chosen counties and districts. Afterwards we selected six villages from per chosen township and six communities from per chosen street randomly. A random sampling method was used to select 30 babies born during 2010–2013 and their mothers in every chosen village, and, 60 in every sampled community. Expected sample size of our study was approximately 32,400 infants and their mothers. But 2373 subjects were unwilling to join in the study (response rate: 92.68%). Therefore 28,644 single live infants were chosen for this project. And 481 objects were removed for unknown birth weight and childbearing history. Moreover, 812 subjects were removed who had more than 3 children. In the end, a total of 27,351 singleton live infants were selected.

### Data collection

All data was stated by the mothers of the chosen children, including socio-demographical information and information on maternal lifestyles during pregnancy. Xi’an Jiaotong University Health Science Center devised all questionnaires. Ten field teams that every team comprised 10–12 members were formed for these counties or districts. As soon as completing every questionnaire, the supervisors were responsible for detecting any errors and/or imperfect information. All data collection was completed in the local village clinics and community health service centers. Our study was sustained by the local hospitals and health administrative departments as well as the Shaanxi province Ministry of Health.

### Study variables

Controlling for potential confounding factors was necessary when determining the relationship parity and macrosomia. Based on the currently available body of knowledge and the nature of our data, we selected potential confounding factors from three groups of variables: children, family and mothers. The factors included within the children group were the child’s sex and gestational age. The factors included within the family group were economic conditions, region and residence. The factors included within the mother group were maternal education level, age at the child’s birth, occupation and gestational diabetes.

Primipara: A woman who has borne only one living child.

Multipara: A woman who has given birth to 2 living children.

### Statistical analysis

A database was designed by EpiData version 3.02, and data entry was duplicated. Firstly, the characteristics of participants were summarized using means±SDs for normally distributed continuous variable. The categorical variables were described using count and proportions. The *χ*^*2*^ test was used to prove differences in proportions between groups. On account of the multilevel hierarchical structure of the data, the generalized linear mixed model approach was used, which is a good method for analyzing data with a hierarchical structure and can be applied in sampling investigations. Ultimately, a 2-level analysis was performed to adjust for the effect of randomization by counties/districts and to analyze the associations between parity and macrosomia with county/districts to level 2 and individual to level 1 by nine potential confounding factors. Model 1 adjusted for gestational age and sex of infants. Model 2 adjusted for the variables in model 1 plus the relevant maternal characteristics, including maternal education level, age at the child’s birth, occupation and gestational diabetes. Model 3 adjusted for the variables in model 2 and the status of family characteristics (including economic conditions, region and residence). All statistical analyses were performed using SAS 9.3 (SAS Institute Inc., Cary, NC). Two-tailed *P* < 0.05 was considered statistically significant.

## Result

### Baseline characteristics of the participants

Of the infants, boys accounted for 53.92% of total infants. Amongst the region of the infants, infants in Central Shaanxi, Northern Shaanxi and Southern Shaanxi accounted for 54.41, 25.06 and 20.53% respectively. The childbearing age of mothers was 26.92 ± 4.65 years and approximately 39.45% of them were educated beyond senior high school. The other details of the sample and distribution of the major demographic variables are shown in Table 1.

**Table 1:** Characteristics of the study population

Baseline characteristics	Primipara	Multipara	*P*
Maternal age, year^a^	25.19 (3.69)	29.60 (4.71)	<0.001
Gestational age, week^a^	39.65 (1.29)	39.64 (1.24)	0.549
Sex of infants, n (%)			
Boy	8789 (52.70)	5959 (55.82)	<0.001
Girl	7887 (47.30)	4716 (44.18)	
Season of birth, n (%)			
Spring	4237 (25.41)	2803 (26.26)	<0.001
Summer	3878 (23.25)	2669 (25.00)	
Fall	4166 (24.98)	2595 (24.31)	
Winter	4395 (26.36)	2608 (24.43)	
Region, n (%)			
Central Shaanxi	9513 (57.05)	5367 (50.28)	<0.001
Northern Shaanxi	3750 (22.49)	3105 (29.09)	
Southern Shaanxi	3413 (20.46)	2203 (20.63)	
Mother’s education, n (%)			
Primary school or less	917 (5.51)	2095 (19.67)	<0.001
Junior high school	6992 (42.03)	6524 (61.25)	
Senior high school	4044 (24.31)	1559 (14.64)	
College and above	4683 (28.15)	473 (4.44)	
Residence, n (%)			
Urban area	4643 (27.95)	1135 (10.68)	<0.001
Rural area	11,968 (72.05)	9492 (89.32)	
Mother’s occupation, n (%)			
Peasant/Housework	8932 (53.56)	8275 (77.52)	<0.001
Other	7744 (46.44)	2400 (22.48)	
Household Wealth Index, n (%)			
Poorest	3302 (22.10)	2903 (29.13)	<0.001
Middle	7765 (51.96)	5605 (56.25)	
Richest	3876 (25.94)	1456 (14.62)	

^a^Reported as mean and SD (standard deviation)

### Status of neonatal birth weight

The average birth weight of 27,351 newborns was 3267.21 ± 455.90 g. The neonatal average birth weight of the primipara was 3262.18 ± 452.36 g, and that of the multipara was 3275.07 ± 461.29 g. Neonatal birth weight of the multipara was higher than that of the primipara, and the difference was statistically significant. The incidence of low birth weight infants was 3.38%, and the incidence rate of macrosomia was 6.79%. Among the 27,351 women of childbearing age surveyed, 16,677(60.97%) women were primiparas, and 10,675(39.03%) were multiparas. The birth weight status of the children for primiparas and multiparas were shown in Table 2. The incidence of macrosomia in multiparas was higher than that in primiparas, and the difference was statistically significant (*P* < 0.001).

**Table 2:** Neonatal birth weight of the primiparas and multiparas

Pregnant women	N	Normal	Low birth weight	Macrosomia
Primipara	16,676	15,055 (90.28)	560 (3.36)	1061 (6.36)
Multipara	10,675	9515 (89.13)	364 (3.41)	796 (7.46)
Total	27,351	24,570 (89.83)	924 (3.38)	1857 (6.79)
*χ*^*2*^			0.05	12.31
*P*			0.817	<0.001

### Univariate analysis of possible influencing factors of macrosomia

Univariate analysis results showed that maternal age < 25 years, peasant/housework, living in rural areas and female infants were the protective factors of macrosomia. And the possibility of having a macrosomic infant also increased with gestational age, maternal education level, household wealth index, living in Central Shaanxi and gestational diabetes. Compared with primipara, multipara was also associated with an increased risk of delivering a macrosomic infant (Table 3).

**Table 3:** Univariate analysis of possible influencing factors of macrosomia

Variables	*β*	*Standard Error*	*Waldχ*^*2*^	*P*	*OR* (95% *CI*)
Maternal age					
Y < 25	−0.422	0.057	54.138	<0.001	0.656 (0.586, 0.734)
25 ≤ Y < 30	Ref				
Y ≥ 30	0.047	0.060	0.615	0.433	1.048 (0.932, 1.180)
Gestational age					
W < 37	−0.983	0.228	18.584	<0.001	0.374 (0.239, 0.585)
37 ≤ W < 42	Ref				
W ≥ 42	0.414	0.129	10.272	0.001	1.513 (1.175, 1.950)
Region					
Southern Shaanxi	Ref				
Central Shaanxi	0.155	0.064	5.799	0.016	1.167 (1.029, 1.324)
Northern Shaanxi	0.097	0.074	1.751	0.186	1.102 (0.954, 1.274)
Mother’s education					
Junior high school and below	Ref				
Senior high school	0.138	0.062	4.992	0.025	1.148 (1.017, 1.297)
College and above	0.428	0.059	53.505	<0.001	1.534 (1.368, 1.721)
Household Wealth Index					
Poorest	Ref				
Middle	0.180	0.065	7.597	0.006	1.197 (1.053, 1.361)
Richest	0.423	0.074	32.368	<0.001	1.527 (1.320, 1.766)
Residence					
Urban area	Ref				
Rural area	−0.435	0.054	65.737	<0.001	0.647 (0.582, 0.719)
Mother’s occupation					
Peasant/Housework	−0.238	0.049	23.820	<0.001	0.788 (0.717, 0.867)
Other	Ref				
Parity					
Primipara	Ref				
Multipara	0.170	0.049	12.290	<0.001	1.186 (1.078, 1.304)
Gestational diabetes					
No	Ref				
Yes	1.139	0.321	12.619	<0.001	3.123 (1.666, 5.855)
Sex					
Boy	Ref				
Girl	−0.602	0.051	139.141	<0.001	0.548 (0.496, 0.605)

### Analysis of the relationship parity and macrosomia

**Fig. 1:**
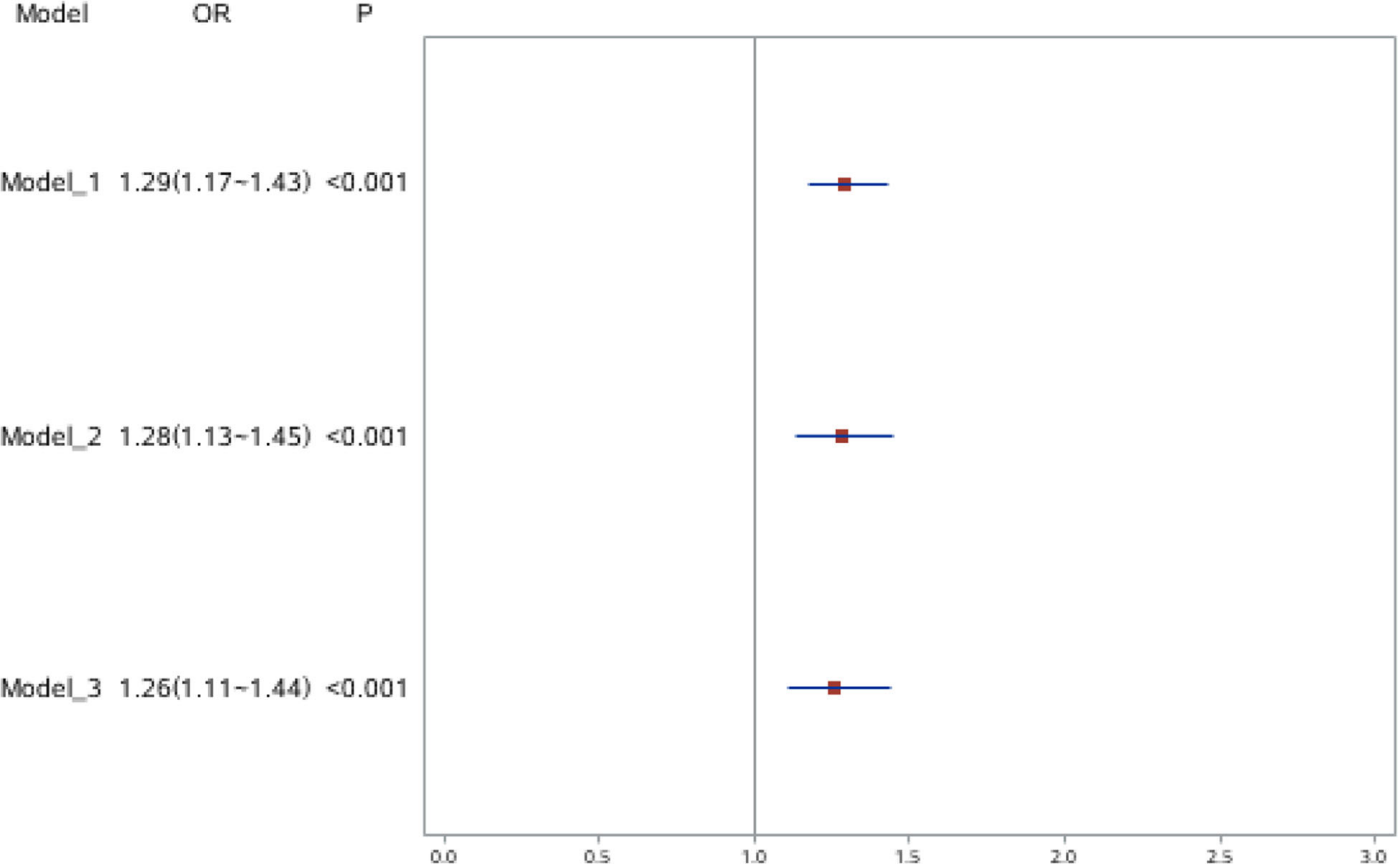
This figure illustrates the odds ratio and 95% confidence intervals of the association between parity and macrosomia using GLMM. GLMM = Generalized linear mixed models. # Model adjustments: model1: adjusted for gestational age and sex of infants. Model 2: adjusted for the variables in model 1 plus mother’s relevant characteristics, including mother’s age, education level, occupation and gestational diabetes. Model 3: adjusted for the variables in model 2 and for status of family characteristics (including economic conditions, region and residence)

In Fig. 1, the GLMM results obtained for macrosomia as an outcome variable represent the association between parity and macrosomia. After adjusting for statistically significant factors in univariate analysis from model 1 to model 3, the odds ratio (OR) showed that the risk of macrosomia in multipara is higher than in primipara. Furthermore, the 3 odds ratios were similar, which indicated that the models were stable. Specifically, a statistically significant association between parity and macrosomia was found. The risk of being born macrosomia was 1.26 times higher for a multipara compared to that for a primipara.

## Discussion

### Main findings

We found that parity was related to the occurrence of macrosomia. The incidence of macrosomia of multipara was higher than primipara, and the difference was statistically significant. After adjusting for statistically significant factors in univariate analysis, analysis based on generalized linear mixed models revealed that the risk of macrosomia was 1.26 times higher for a secondborn child compared to a first born.

### Data interpretation and comparisons with previous studies

The association between parity and macrosomia has been previously investigated in a few studies conducted elsewhere, and increased parity is associated with higher risk of fetal macrosomia [23, 24]. Multiparity is one of the most important risk factors for macrosomia, according to the American College of Obstetricians and Gynecologists’ Committee on Practice Bulletins—Obstetrics [26]. The rate of fetal macrosomia in multiparous women has been shown to be 2–3 times higher than that in control group in the majority of studies [27]. Faith Agbozo. etal noted that the parity of two to three children was related to raised risk for macrosomia [28]. The aforementioned studies illuminated the relationship between parity and macrosomia and provided some references and evidences for our research.

The present cross-sectional study indicated parity of two children was associated with increased risk for fetal macrosomia. Compared to primiparas, multiparas should far strengthen the pre-pregnancy education and the guidance during pregnancy to control pre-pregnancy body mass index and pregnancy weight, and keep the appropriate exercise and balanced diet in order to reduce the incidence of macrosomia.

### Strengths and limitations

The primary strength of the present analysis is the large sample size (27,351 single live births occurring from 2010 to 2013), which accounted for ~ 9% of neonates in Shaanxi Province [29]. Therefore, our results can be generalized to the entire province as well as Northwest China. Another strength of this study is that the birth weight data collected from birth certificates was accurate to the nearest 10 g. Moreover, the generalized linear mixed models adjusted for relevant covariates were generated to further elucidate the association between parity and macrosomia. Limitations of our data should also be noted. Some major confounders, including pre-pregnancy BMI, diet, weight gain during pregnancy and so on, were not adjusted for because we lacked these data [30, 31]. Nevertheless, the current study is the first and largest survey that has presently been conducted in Northwest China, and provides the best information on the relationship between parity and macrosomia in this geographical region.

## Conclusions

The present cross-sectional study indicated parity of two children was associated with increased risk for macrosomic births compared with parity of one child. Compared to primiparas, multiparas should far strengthen the pre-pregnancy education and the guidance during pregnancy to control pre-pregnancy body mass index and pregnancy weight, and keep the appropriate exercise and balanced diet in order to reduce the incidence of macrosomia.

## Data Availability

The datasets generated and/or analysed during the current study are not publicly available. Because the data collection has been completed together by the research team, and the team has contributed a lot of effort for it. And it is available from the corresponding author on reasonable request.
